# Severe Hypoglycemia Caused by a Giant Borderline Phyllodes Tumor of the Breast: A Case Report and Literature Review

**DOI:** 10.3389/fendo.2022.871998

**Published:** 2022-05-26

**Authors:** Yaoxia Liu, Min Zhang, Xudan Yang, Min Zhang, Zhen Fan, Yi Li, Tao Wang, Ping Chen

**Affiliations:** ^1^ Department of Geriatrics, Sichuan Provincial People’s Hospital, University of Electronic Science and Technology of China, Chengdu, China; ^2^ Chinese Academy of Sciences, Sichuan Translational Medicine Research Hospital, Chengdu, China; ^3^ Department of Pathology, Sichuan Provincial People’s Hospital, University of Electronic Science and Technology of China, Chengdu, China; ^4^ Mastology Department, Sichuan Provincial People’s Hospital, University of Electronic Science and Technology of China, Chengdu, China; ^5^ Department of Pediatrics, West China Second University Hospital, Sichuan University, Chengdu, China; ^6^ Key Laboratory of Birth Defects and Related Diseases of Women and Children (Sichuan University), Ministry of Education, Chengdu, China; ^7^ The Cardiac Development and Early Intervention Unit, West China Institute of Women and Children’s Health, West China Second University Hospital, Sichuan University, Chengdu, China

**Keywords:** phyllodes tumor, breast, hypoglycemia, non-islet cell tumor hypoglycemia, insulin-like growth factor-2

## Abstract

A case of hypoglycemic coma caused by a giant borderline phyllodes tumor of the breast has been described. The patient, a 63-year-old woman, was admitted with recurrent unconsciousness. She had a giant breast tumor with decreased blood glucose, insulin, and C-peptide. The patient’s hypoglycemia resolved rapidly after resection of the breast tumor. Pathological examination indicated a borderline phyllodes tumor of the breast, and immunohistochemistry suggested high expression of insulin-like growth factor-2 (IGF-2) in the tumor tissue. A literature review is also included to summarize the clinical characteristics of such patients and to serve as a unique resource for clinical diagnosis and treatment of similar cases.

## Introduction

Hypoglycemia is an endocrine emergency, which can manifest as impaired consciousness or even death in severe cases. Hypoglycemia is frequently caused by improper antidiabetic drug use or insulin overproduction, such as islet cell tumors (1, 2). Non-islet cell tumor hypoglycemia (NICTH) is very rare (3). The most common cause of hypoglycemia of this type is tumoral overproduction of incompletely processed insulin-like growth factor-2 (IGF-2), which stimulates insulin receptors and increases glucose utilization (3, 4). Other potential but less common causes include the production of autoantibodies against insulin or the insulin receptor and extensive tumor burden destroying the liver or adrenal glands (4). NICTH occurs more commonly in patients with mesenchymal tumors, fibromas, carcinoids, myelomas, lymphomas, and hepatocellular and colorectal carcinomas (3, 4). It is very rare that a phyllodes tumor of the breast causes NICTH. Breast phyllodes tumors that cause NICTH are extremely uncommon. PTBs (phyllodes tumors of the breast) are rare fibroepithelial tumors that make up about 0.5% of all breast tumors (5, 6). Histologically, PTBs are classified as benign, borderline, or malignant, and borderline tumors account for only 12%–18% of cases (7). We present here a case of a giant borderline phyllodes tumor of the breast causing NICTH in which hypoglycemia disappeared after mastectomy, and immunohistochemistry confirmed that the tumor expressed high amounts of IGF-2.

## Case Presentation

A 63-year-old woman was taken to the emergency room due to unconsciousness around 6:00 a.m. in April 2016. A blood examination showed severe hypoglycemia (1.4 mmol/L). A total of 40 ml of a 50% glucose solution and 500 ml of 10% glucose solution were intravenously administered to the patient, which restored her serum blood sugar level (9.5 mmol/L) and consciousness. Subsequently, she was discharged from the hospital the same day. The patient lost consciousness again 6 days later (at 6:00 a.m.) and was taken to the emergency department. She received another glucose solution infusion due to hypoglycemia (1.9 mmol/L), which alleviated her hypoglycemic symptoms. According to her medical history, a bean-like hard mass was found in the right breast 1 year ago, which gradually increased to the size of a soccer ball, accompanied by redness of the skin, pinprick-like pain, and nipple ulceration. The patient was diagnosed with “nasopharyngeal carcinoma” 20 years ago, and there was no recurrence after radiotherapy. She denied a history of hypoglycemic drug use and a history of poor appetite and wasting.

Physical examination after admission revealed the following: no enlargement of superficial lymph nodes and a BMI of 22.5 kg/m^2^. The right breast was large. The mass was soft and uneven in texture. Skin temperature was significantly elevated, and nipples were cauliflower-like with erosion. Blood examination showed hypokalemia (3.08 mmol/L) and a normal glycosylated hemoglobin level (5.4%). Thyroid hormone values were as follows: FT3 = 2.53 pmol/L (Ref. = 2.63–5.70 pmol/L), FT4 = 6.56 pmol/L (9.01–19.05 pmol/L), and TSH = 6.209 MIU/L (0.35–4.94 MIU/L). The blood routine, liver and kidney function, and tumor markers were normal, and insulin antibody (IAA) was negative.

The patient was given a continuous 10% glucose solution (40 ml/h) intravenously, as well as thyroid hormone supplementation (Levothyroxine Sodium Tablets 25 μg qd) and potassium supplementation. The patient still appeared drowsy and unresponsive at 7:00 am on the second day of admission, and blood glucose was measured at 2.2 mmol/L. Immediately, she was given 50% glucose solution, 40 ml of intravenous injection, and an accelerated glucose drip rate (60 ml/h). After that, the blood glucose fluctuated between 2.9 and 4.5 mmol/L. During the hypoglycemic episode, blood tests revealed severe hypo-insulinemia (<0.1 μU/ml), low C peptide (0.09 ng/ml), low insulin release index (<0.002), low GH (0.075 ng/ml), and normal IFG1 levels. Other hormonal indicators suggested normal pituitary–adrenal axis and pituitary–sex hormone axis.

**Figure 1:**
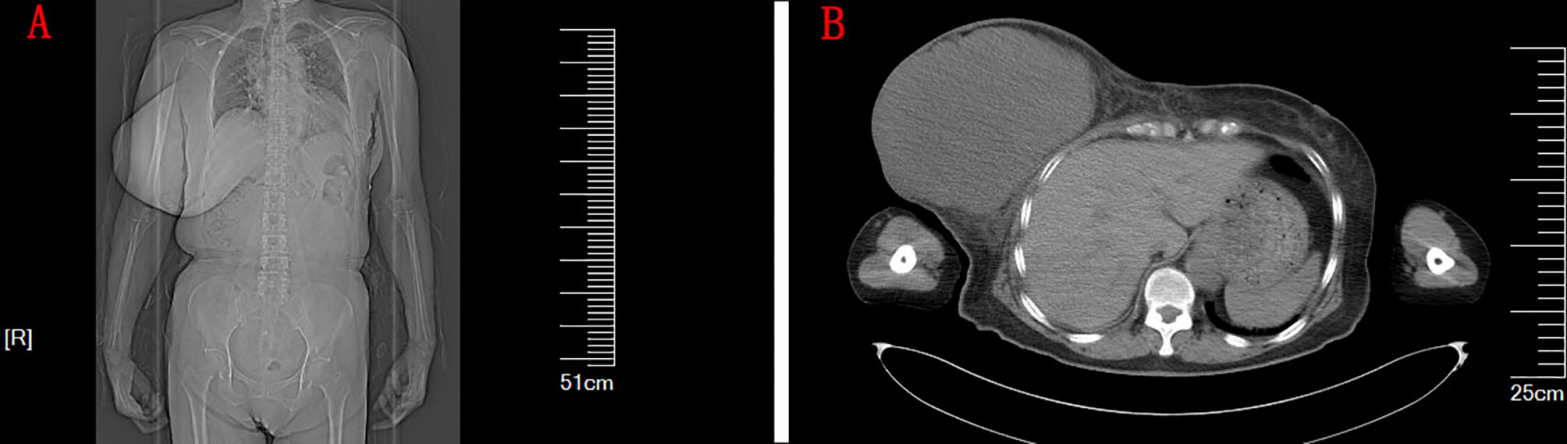
**(A)** The CT image of the patient’s chest and abdomen. **(B)** The CT of the chest. A large soft tissue density mass was seen in the right breast, measuring approximately 17.1 cm × 13.2 cm × 17.2 cm, with clear borders, regular morphology, and uniform density.

There was no abnormality in the nasopharynx on CT of the head and chest (**Figures 1A, B**). A large soft tissue density mass was seen in the right breast, measuring approximately 17.1 cm × 13.2 cm × 17.2 cm, with clear borders and regular morphology, and no enlarged lymph nodes were seen in the bilateral axillae. The breast ultrasound showed a huge hypoechoic mass with an irregular liquid dark area in the right breast and an enlarged right nipple. Ultrasound of lymph nodes showed no enlargement of cervical, axillary, and inguinal lymph nodes. Mammography showed a large occupying lesion in the right breast. A puncture biopsy of a breast tumor confirmed fibroepithelial tumor.

**Figure 2:**
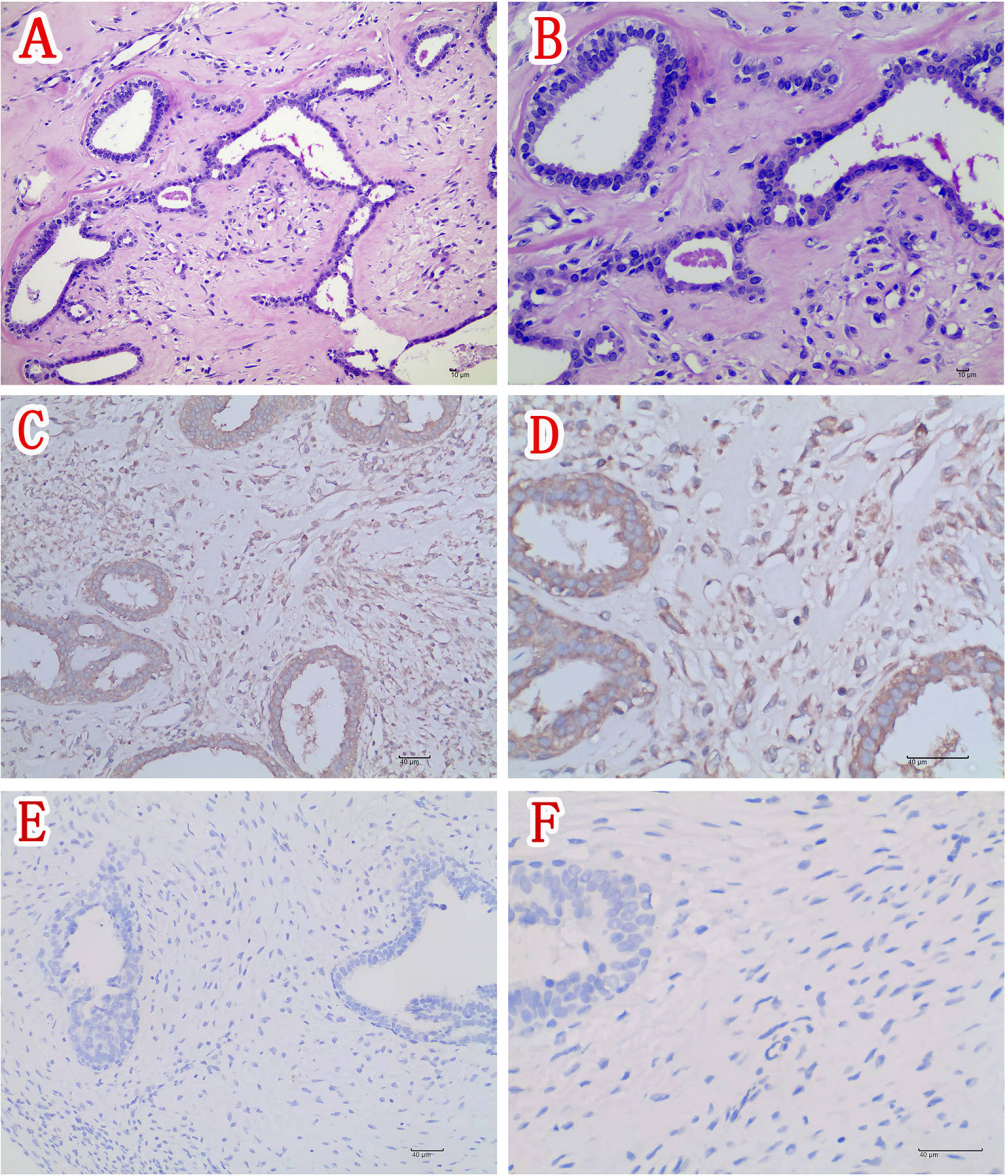
**(A)** Section of the tumor showing tumor mesenchymal cell hyperplasia and unremarkable ductal epithelial hyperplasia (H&E ×200). **(B)** Higher magnification of section in panel **(A)** showing moderate atypia of mesenchymal cells (H&E ×400). **(C)** Immunohistochemical staining of this phyllodes tumor (IGF-2 IHC ×200) and **(D)** higher magnification of the phyllodes tumor depicted in panel **(C)** showing positivity for IGF-2 in the cytoplasm of tumor mesenchymal cells and ductal epithelium (IGF-2 IHC ×400). **(E)** Immunohistochemical staining of the control phyllodes tumor of breast obtaining from another woman without hypoglycemia (IGF-2 IHC ×200) and **(F)** Higher magnification of the phyllodes tumor depicted in Figure 2E showing negativity for IGF-2 in the control tumor tissue (IGF-2 IHC ×400).

A right mastectomy and resection of a large mass in the right chest wall were performed 1 week after admission. Postoperative paraffin pathology confirmed the right breast borderline phyllodes tumor (**Figures 2A, B**) with a maximum diameter of approximately 19 cm. The nipple had no tumor involvement. Postoperative immunohistochemical staining was as follows: ER epithelial (+), PR epithelial (+), HER2 (-), CD117 (-), CD34 (-), P53 (+), CK5/6 (-), and Ki-67 about 2%. Immunohistochemical staining of the phyllodes tumor with rabbit polyclonal anti-IGF-2 antibodies showed immunopositivity for IGF-2 in the cytoplasm of tumor mesenchymal cells and ductal epithelium (**Figures 2C, D**).

There were no hypoglycemic episodes after the postoperative glucose infusion was stopped. The thyroid function was normal on recheck before discharge. Long-term follow-up to date (5 years) revealed that the patient had no hypoglycemic episodes and no breast tumor recurrence.

## Discussion

The patient had recurrent nocturnal and early morning hypoglycemia, and the blood insulin and C-peptide were significantly lower (insulin < 0.1 μU/ml, C-peptide 0.09 ng/ml) during the hypoglycemic episodes, suggesting that the patient’s hypoglycemic episodes were not mediated by insulin. The patient had no history of hypoglycemic drug use and alcohol abuse, had normal liver and kidney function, and had no cachexia. As a result, the etiology of the patient’s hypoglycemia was investigated to see if it was due to a lack of elevated blood sugar hormones or hypoglycemia caused by tumor secretion of insulin-like growth factors in non-islet cell tumors. There are many types of elevated blood sugar hormones, including glucagon, glucocorticoids, catecholamines, growth hormone, and lactogen. Thus, except for hypopituitarism or adrenal cortical crisis, a single hormone deficiency rarely causes hypoglycemia (1, 2). In the patient, the hypopituitary–adrenal function, IGF-1, and PRL levels were normal. As a result, hypoglycemia due to a lack of multiple glucagon hormones was uncommon, even when the patient also had hypothyroidism. This patient had recurrent episodes of hypoglycemia despite the continuous infusion of glucose before the removal of the breast tumor. After surgical removal of the breast tumor, there were no further episodes of hypoglycemia, and glucose infusion was discontinued. As a result, the phyllodes tumor of the breast was confirmed as the cause of the patient’s hypoglycemia. The hypoglycemia was thought to be caused by the tumor’s overproduction of IGF-2 because this phyllodes tumor tissue was immunopositive to the protein. However, serum IGF-2 levels were not measured before and after surgery, which limited the study’s findings.

The most common cause of NICTH is the overproduction of incompletely processed IGF-2 by the tumor, which stimulates insulin receptors and increases glucose utilization (3, 4). This type of incompletely processed IGF-2 is also called big-IGF-2. Big-IGF-2 is highly homologous to insulin, with its pro-fragments B, C, and A corresponding to similar structures of insulinogenic B chain, C peptide, and A chain, respectively (3). They can bind to the insulin receptor and cause hypoglycemia, as well as mediate the intracellular transfer of potassium, resulting in hypokalemia (3). Hypoglycemia caused by big-IGF-2 inhibits endogenous insulin secretion, resulting in a significant drop in insulin levels and C peptides in the blood of the patient (3).

Due to the high structural homology, big-IGF-2 can also bind to the IGF-1 receptor family (3). IGF-1 is an effector of growth hormone and is secreted by the liver to exert growth hormone pro-growth developmental effects. An increase in large IGF-2 can lead to limbic hypertrophy-like manifestations and can also stimulate rapid growth and proliferation of the tumor itself. On the other hand, it can negatively feedback inhibit IGF-1 and GH secretion, resulting in lower IGF-1 and GH levels (3). However, the molecular weight of big-IGF-2 is different from mature IGF-2; a normal mature IGF-2 has a molecular weight of 1.5 kDa, whereas the tumor produces big-IGF-2 with a much larger molecular weight, between 10 and 20 kDa (3). As a result, different bands may appear on expression of IGF-2 protein in serum or tumor tissue by Western immunoblot (3).

This is a rare case of NICTH. NICTH occurs commonly in patients with mesenchymal tumors, fibromas, carcinoid, myelomas, lymphomas, hepatocellular, and colorectal carcinomas (3, 4). Breast phyllodes tumors are rarely present.

We performed a comprehensive search of databases (English and Chinese Language), including PubMed, Web of Science, Embase, the Cochrane Library, SinoMed, and CNKI, from the creation of the database up to February 12, 2021. A literature search through databases revealed 12 cases (**Table 1**) of NICTH due to phyllodes tumor of the breast (5, 8–18), including two benign (12, 14), three borderline (5, 8, 9), and six malignant (15–17) PTBs (one unknown) (10). All the patients were female, aged 20–54 years, and the patient, in this case, was the oldest. The duration of their breast tumors ranged from 3 months to 20 years, and all of the tumors were giant breast tumors. Hypoglycemia was manifested in these patients as impaired consciousness, with some patients experiencing recurrent hypoglycemia despite receiving continuous intravenous glucose solution (11–15, 18). The patients often combined with hypokalemia (5, 11, 13, 14, 16, 18). Serum IGF-2 concentration was normal (5, 11), decreased (16, 17), or elevated (18), and big-IGF-2 was seen in serum Western immunoblot analysis (WB) (9, 13–15). In some cases, IGF-2 detection was carried out in tumor tissues. In these tumor tissues, increased IGF-2-mRNA expression was detected by polymerase chain reaction (PCR) (5, 17), and big-IGF-2 protein was detected by WB (9). In some cases, IGF-2 showed immunopositivity in pathological tumor specimens by immunohistochemical staining (8, 9, 17). There was also a case in which the tumor secreted insulin ectopically (16).

**Table 1:** Characteristics of reviewed reports of non-islet cell tumor hypoglycemia due to phyllodes tumor of breast.

Authors	Reported year	Sex	Age (years)	Tumor course	Tumor weight (kg)	Tumor size (cm)	Symptoms of hypoglycemia	Glu (mg/dl)	Subtype of tumor	Blood IGF-2		Tissue IGF-2				
										Concentration	WB*	Concentrations	PCR†	WB**	IHC ‡
Zhao et al. (8)	2021	F	45	6 M	N/A	25	Unconsciousness	14	Borderline	N/A	N/A	N/A	N/A	N/A	+
Hikichi et al. (9)	2018	F	50	1 Y	4.5	27×23×23	Unconsciousness	14	Borderline	N/A	Big-IGF-2	N/A	N/A	Big-IGF-2	+
Saito et al. (5)	2016	F	48	N/A	>5	25×18×17	Unconsciousness	20	Borderline	High	N/A	High	N/A	N/A	N/A
Sharma et al. (10)	2016	F	37	N/A	N/A	25×20×20	Dizziness	16	N/A	N/A	N/A	N/A	N/A	N/A	N/A
Pacioles et al. (11)	2014	F	51	3M	N/A	29.5×26×14	Unconsciousness	16	Malignant	Normal #	N/A	N/A	N/A	N/A	N/A
Agrawa et al. (12)	2013	F	20	N/A	N/A	34	Unconsciousness	20	Benign	N/A	N/A	N/A	N/A	N/A	N/A
Renard et al. (13)	2012	F	49	1 Y	5.8	27×26×20	Unconsciousness	24	Malignant	N/A	Big-IGF-2	N/A	High	N/A	N/A
Hino et al. (14)	2010	F	49	2 Y	4.2	25×20×20	Unconsciousness	21	Benign	N/A	Big-IGF-2	N/A	N/A	N/A	N/A
Aguia et al. (15)	2007	F	52	4 Y	10	33	Unconsciousness	12	Low-grade PTB	N/A	Big-IGF-2	N/A	N/A	N/A	N/A
Herr et al. (16)	2004	F	33	3 W	N/A	18×16.5×11.5	Unconsciousness	24	Malignant	Low &	N/A	N/A	N/A	N/A	N/A
Kataoka et al. (17)	1998	F	54	20 Y	9	35×28×27	Unconsciousness	18	Malignant	Low	N/A	High	N/A	NA	+
Li et al. (18)	1983	F	43	2 Y	4.2	28×18×15	Unconsciousness	10	Malignant	High@	N/A	N/A	N/A	N/A	N/A
This case	–	F	63	1 Y	N/A	19	Unconsciousness	25.2	Borderline	N/A	N/A	N/A	N/A	N/A	+

*Expression of IGF-2 protein in serum by Western immunoblot. †Expression of IGF-2 mRNA in the tumor tissue by PCR. **Expression of IGF-2 protein in the tumor tissue by Western immunoblot. ^‡^Immunohistochemical staining of IGF-2 in the tumor tissue. +Positive. #In this case, the concentration of IGF-2 in serum was normal, but the IGF-II/IGF-I ratio was elevated. &In this case, the concentration of IGF-2 in serum was low, but insulin level was elevated, because of the tumor ectopic-secreted insulin. @In this case, plasma levels of insulin-like protein were increased, but it was not determined whether the insulin-like protein was IGF-2 or not.

N/A, not available.

In all cases, continuous intravenous glucose infusion was used to treat hypoglycemia. Two of these cases (10, 11) combined oral and intravenous glucocorticoids. The prognosis of phyllodes tumor of the breast causing NICTH is related to the malignancy of the tumor. In the majority of cases, hypoglycemia remission was achieved after mastectomy of the breast tumor, except for one patient (15) with low-grade PTB who eventually died of aspiration pneumonia and renal failure and one patient with metastatic breast malignancy (13).

Treatment for hypoglycemia caused by a non-islet cell tumor should include immediate correction of the hypoglycemia as well as prompt tumor resection. Increased caloric intake (sometimes through enteral or parenteral nutrition) and intravenous glucose or dextrose administration if necessary are used to treat NICTH in inoperable patients (3, 4). Glucocorticoids have been found to increase the clearance of large IGF-2, and glucocorticoid therapy (prednisone 30–60 mg/day) is an option for patients with untreatable malignancies (4). If hypoglycemia persists, patients whose blood glucose levels respond to glucagon therapy may be given long-term intravenous glucagon infusion (0.06–0.30 mg/h) or growth hormone may be added (4). However, because of the risk of pro-tumor growth, growth hormone is generally not chosen except to relieve the suffering in patients with NICTH end-stage cancer (4).

We conclude from this patient’s case and the literature that in patients with hypoglycemia due to phyllodes tumor of the breast, a continuous high-concentration glucose infusion is required preoperatively to avoid recurrent hypoglycemia caused by big-IGF-2, and aggressive surgical removal of the breast lesion is also required.

## Conclusion

This case reports a rare, hypoglycemic coma due to a borderline phyllodes tumor of the breast. The patient’s hypoglycemia resolved rapidly after the removal of the breast tumor. Pathological examination confirmed a borderline phyllodes tumor of the breast, and immunohistochemical staining showed high expression of IGF-2 in the tumor tissue. According to research, these patients frequently have severe hypoglycemia, impaired consciousness, a giant breast tumor, and detectable big-IGF-2 in serum or tumor tissue. Hypoglycemia resolved rapidly after resection of the tumor, and the prognosis depends on the malignancy of the tumor.

## Data Availability Statement

The original contributions presented in the study are included in the article/supplementary material. Further inquiries can be directed to the corresponding authors.

## Ethics Statement

The patient has signed the informed consent and fully acknowledged the details of examinations and inspection items.

## Author Contributions

YLiu was involved in the study concept, study design, and manuscript preparation. MZ (2nd author) carried out the definition of intellectual content and manuscript review. XY handled data analysis and statistical analysis. MZ (4th author) carried out data acquisition. ZF and YLi conducted the clinical studies. TW was involved in the literature research and manuscript editing. TW and PC performed the role of guarantors for the integrity of the entire study. All authors contributed to the article and approved the submitted version.

## Funding

This article was supported by the National Natural Science Foundation of China (No. 81701888), the Science and Technology Program of Sichuan (2019YFS0239), and the Health and Family Planning Commission Foundation of Sichuan Province (No. 17PJ262).

## Conflict of Interest

The authors declare that the research was conducted in the absence of any commercial or financial relationships that could be construed as a potential conflict of interest.

## Publisher’s Note

All claims expressed in this article are solely those of the authors and do not necessarily represent those of their affiliated organizations, or those of the publisher, the editors and the reviewers. Any product that may be evaluated in this article, or claim that may be made by its manufacturer, is not guaranteed or endorsed by the publisher.
